# The HI HOPES data set of deaf children under the age of 6 in South Africa: maternal suspicion, age of identification and newborn hearing screening

**DOI:** 10.1186/s12887-016-0574-1

**Published:** 2016-03-22

**Authors:** Claudine Störbeck, Alys Young

**Affiliations:** Centre for Deaf Studies, University of the Witwatersrand, Wozani Building, Education Campus, 27 St Andrew’s Road, Parktown, Johannesburg, South Africa; School of Nursing, Midwifery and Social Work, University of Manchester, Jean McFarlane Building, Oxford Road, Manchester, UK

**Keywords:** Newborn hearing screening, Maternal suspicion, Age of identification, Deaf infants, South Africa

## Abstract

**Background:**

Identification of deafness before 3 months of age substantially improves the socio-linguistic and cognitive development of deaf children. Existing studies demonstrating the feasibility of newborn hearing screening in South Africa have used small samples unrepresentative of general population characteristics. This study establishes the characteristics of the largest data set of deaf infants and their families in South Africa on which there is baseline and longitudinal data (*n* = 532); explores its representativeness in terms of socio-demographic features and reports on access to and quality of newborn hearing screening within the sample. It examines specifically the relationship between age of maternal suspicion of childhood deafness and age of identification of deafness by cohort characteristics.

**Methods:**

Secondary analysis, using descriptive and inferential statistics, of a pre-existing longitudinal data set (*n* = 532) of deaf infants under 6 years of age, and their families, collected as routine monitoring of the HI HOPES (HH) early intervention programme.

**Results:**

The HH cohort is representative in terms of racial profile and private/public health care use but displays slightly higher level of maternal education and slightly lower socio-economic status than national comparators. 102 out of 532 infants had undergone newborn hearing screening, resulting in 29 true positives, 15 of whom would have met the criteria for targeted screening. Later onset deafness does not account for the 73 false negatives. The median age of maternal suspicion (*n* = 247) of infant deafness was 18 months; the median age of identification of 28 months. Age of identification was unrelated to private/public health care status. The median delay between age of suspicion and age of identification was significantly longer in the public sector (7 m; IQR 0–15 m) compared to the private sector (2 m; IQR 0–8.5 m) (*p* = 0.035). Age of suspicion was unrelated to level of maternal education. Earlier age of suspicion did not predict earlier identification.

**Conclusion:**

Targeted screening as timely response to maternal suspicion offers a viable means to reduce substantially the age of identification of deafness in South Africa until implementation of newborn hearing screening on a population-wide basis can be justified.

## Background

The developing world accounts for 90 % of all deaf children, estimated to be 32 million, with the greatest prevalence (70 %) in South Asia, Asia Pacific, and Sub-Saharan Africa [32]. High resource countries of the developed world have invested in universal newborn hearing screening, early diagnosis of deafness, and comprehensive early intervention services based on evidence of the major linguistic, cognitive and socio-emotional advantages that result [13]. In Sub-Saharan Africa in general, early detection of deafness in the first few months of life is regarded as much less of a priority than the prevention, detection and treatment of life threatening diseases such as HIV Aids and TB [10]. However, the situation in South Africa is potentially very different.

Although South Africa is part of the developing world, it is an upper middle income country with a reasonably well developed healthcare system including significant quality in the training of audiological professionals and pediatricians. Yet it is estimated that more than 90 % of the 6200 deaf babies born in South Africa annually will not have the prospect of early identification and diagnosis [23, 27]. This is despite high quality audiological training and in some regions the infrastructure to ensure that international best practice standards could be met (particularly in the private health sector). Nonetheless, South Africa has a shortage of ENT specialists and Audiologists [2]. Additionally, there is a reluctance to prioritise the screening and diagnosis process with the necessary budgets to implement these. In part, this reluctance results from the dearth of evidence originating from the specific context of South Africa that might justify its priority alongside other competing health, social and economic demands.

To date, South African studies of the benefits and costs of early hearing detection have been small scale, have not encompassed the full diversity of general population characteristics, have focused on specific clinics and their patients, and have not taken a longitudinal perspective that includes early intervention to promote linguistic, social and cognitive development [4, 5, 8, 25–27, 29]. Although this evidence has demonstrated, for example, that the implementation of a screening programme is possible in both a hospital and a midwife-led obstetric unit, it is not reflective of population level concerns. This is especially important in a country such as South Africa that has 11 official languages, wide socio-economic disparities, a diversity of cultural groups who constitute nationhood, and a health care system where only 16 % of the population has access to private health insurance [14].

In terms of studies that focus on identification, to date we have found only small cohort studies referring to age of identification in South Africa. One study (*n* = 54) at a pre-school in the Western Cape calculated the average age of identification at 23 months [29] two retrospective reviews of patient files– one at a University training clinic and the second at a tertiary level public healthcare facility – found the average ages of identification to be 42.1 months (Range: 2.2 – 128.2, 27.6 SD; *n* = 49) [26] and 44.5 months (Range: 1 – 5.9, *n* = 260) respectively [1]. Finally a retrospective study of CI [Cochlear Implant] candidates, primarily in the private sector with over 66 % of the children being white, recorded the average age of identification as 15.3 months (range 0.5 – 45 months, 9.3 SD; *n* = 121) [7].

Key pieces of missing evidence that might justify a more comprehensive approach to EHDI [Early Hearing Detection and Intervention] in South Africa include large scale studies of deaf infants and their families, samples that are indicative of the full diversity of languages, cultures and socio-economic circumstances, a longitudinal perspective that can track infants’ progress through the health care system over several years, and the prospective monitoring of deaf children’s language outcomes (whether early or late identified).

In what follows, we report on a large scale, cohort study of 532 deaf children and their families from birth to 6 years old in South Africa. We describe the characteristics of the sample and the extent to which it might be regarded as representative of the diversity of the general population. We then focus on one specific concern as a means of uncovering variations in the quality and accessibility of the hearing health care system: namely the relationship between maternal suspicion of deafness, age of identification of deafness, and newborn hearing screening. We explore the influence of the variables of maternal education, private/public health care and child risk factors on age of identification and the gap between maternal suspicion and identification. Finally we discuss the implications of the results for improvements in the current system of hearing detection, regardless of the question of whether universal newborn hearing screening is justified.

## Methods

Structured longitudinal data were collected over a 5-year period (September 2006 – December 2011) on all deaf^1^ children and their families who were enrolled in a home-based early intervention programme in South Africa: HI HOPES (HH) [21, 22]. The High Hopes Programme defines ‘deafness’ as any type or level of hearing loss (from mild to profound), including both unilateral and bilateral deafness.

Launched initially in Gauteng Province in 2006 but later extending to KwaZulu Natal and The Western Cape, the HH programme provides a free early intervention service for deaf children and their families from birth up to 6 years of age. Professionals can refer as well as parents self-refer but there is no automatic referral from health or education services into the programme. Family support and language development interventions are based on the SKI HI curriculum [15] adapted for the South African context. Families receive regular home visits from parent advisors and deaf mentors. The programme is neither biased toward any particular amplification (hearing aids, cochlear implants, bone-anchored devices) nor language and communication approach (signed, spoken, mixed) and the child’s language development is monitored at regular intervals against norm-referenced standards [30].

On entry into the programme a comprehensive, structured history of child and family is taken using a standard reporting form that is updated at regular intervals during the child/family enrollment. Approval for the data to be used for research purposes in pseudo-anonymised form is dependent on individual parent consent and the research study overall was approved by the University of Witwatersrand, Johannesburg research ethics committee. The university also acts formally as the data custodian.

Data were drawn from the original child and family registration form on entry into the HH programme (and updated annually), the individualised child and family intervention plans, and audiological profiles (where available) resulting in 162 variables. All data were entered into a Microsoft Excel database and organized by individual child (pseudo-anonymised). Missing data were requested on an individual basis from the relevant HH early interventionists (parent advisors and mentors) and where necessary the families directly. The accuracy of the database entry was verified through a process of checking every 5^th^ child’s data entry on all data points with a 94.6 % accuracy. Any discrepancies were checked back with the primary data and confirmed, all incomplete data were excluded. Data reported here were analyzed using descriptive and inferential statistical methods.

## Results

### Characteristics and representativeness of the sample

711 infants were referred to HI HOPES between Sept 1^st^, 2006 and December 31^st^ 2011. This represents 3.8 % of an estimated population of 18,553 deaf children born in the three provinces of Gauteng, KwaZulu Natal and the Western Cape during the study period calculated at 5.5/1000 live births [11, 17]. Of the 711 infants referred, 149 did not receive services or had only minimal contact due to inappropriateness of referral (e.g. did not have a hearing loss or were significantly over-age and therefore direct school referral was more appropriate). Of the remaining 562, a further 30 were excluded from the data set reported here because of substantially incomplete data. The final sample on which this paper is based is 532 infants, of these 514 had confirmed bilateral hearing loss, 13 had a unilateral loss and in 5 cases not enough audiological testing had been completed to be certain whether unilateral or bilateral deafness was present. Of the 532 children, 56 % were boys (*n* = 298). In 44 % of cases (228 out of 517 where data are available) the deaf child was the first child in the family. In 34 % of cases (174/522) the deaf child was the only child. There were four sets of twins, of whom only one was deaf in each case. The infants (*n* = 532) were distributed amongst the 3 provinces where HI Hopes operates: Gauteng [GT] (*n* = 337), KwaZulu Natal [KZN] (*n* = 92) and The Western Cape [WC] (*n* = 103). Collectively these three provinces represent 54 % of the South African population [14, 18].

In South Africa four racial categories are used for official purposes, with the child classified according to the father’s race, unless it is a single mother whose racial category would then predominate. Of the 532 infants, 73.7 % are Black, 11.3 % are Coloured, 9.2 % are White and 5.8 % are Indian. This compares well with the Census 2011 national population statistics of 79.2 Black, 8.9 Coloured, 8.9 White and 2.5 % Indian [18]. Each of the provinces has a unique racial and cultural make-up based on its own history and current context within South Africa, and the extent to which the racial characteristics of HH infants reflect those in the three provinces reported on are presented in Table 1: Racial characteristics by province for HH sample (*n* = 532) compared to the overall provincial population [18].

**Table 1:** Racial characteristics by province for HH sample (*n* = 532) compared to the overall provincial population (SSA, [18])

SA race groups	HI HOPES vs SA Provinces	WC % (*n* = 103)	KZN % (*n* = 92)	GT % (*n* = 337)	HH population % (*n* = 532)	SA population %
Black	HI HOPES	53.3	70.7	80.7	73.7	79.2
	Province	32.8	86.8	77.4	65.6	
Coloured	HI HOPES	44.6	2.2	3.6	11.3	8.9
	Province	48.8	1.4	3.5	17.9	
Indian	HI HOPES	0	22.8	2.9	5.8	2.5
	Province	1.0	7.4	2.9	3.7	
White	HI HOPES	1.9	4.3	12.8	9.2	8.9
	Province	15.7	4.2	15.6	11.8	

The HH dataset (*n* = 532) is both nationally and provincially broadly representative, with the exceptions of the Western Cape where the White population is under-served and the Black over-served, and the Indian community in KZN who are over-served in comparison to the overall demographic composition of the province.

Parents were asked to indicate the primary language used in their home. When the mother and father used different languages, both languages were listed. This query resulted in 15 separate languages and 13 language combinations. The vast majority of families, regardless of bi/multi-lingual contexts at home chose to communicate in only one language with their deaf child (95 %; 506/532). This monolingual approach is particularly unusual in a society where it is typical for hearing infants to grow up being exposed to and using multiple languages. Amongst the small minority of HH HOPES children who were being raised bilingually (*n* = 26, 4.8 %) the combination of English/Afrikaans was the most common (*n* = 10).

From a cultural perspective, it was inappropriate to enquire about income or employment as questions about personal financial status are regarded as highly sensitive in South Africa without the full anonymity of a census-type survey. However, access to healthcare and housing [16] are two generic indicators of socio- economic status in South Africa. These were used to explore further the representativeness of the HH sample.

Nationally, approximately 17 % of the population benefit from the private health sector through membership of a medical insurance scheme and 83 % are within the public health sector. A further 10 to 20 % of the national population [3] will access some aspects of the private health care sector through out-of-pocket expenses when they choose to. In the 2011 National General Household survey [19] when respondents were asked where they accessed health care, 70.6 % stated they accessed public health and 27.9 % stated they accessed private healthcare. When considering only the 3 provinces in which HH worked (GT, WC and KZN) the provincial statistics revealed 64.1 % accessed public health but in the HI HOPES sample, 85 % were accessing public health which mirrors more closely the national trend.

**Fig. 1:**
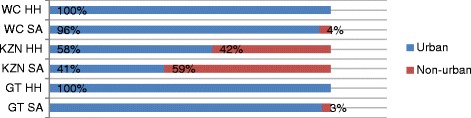
Urban vs Rural breakdown by province for the HH sample (*n* = 532) in comparison to provincial population [20]

In terms of housing, South Africans live in a range of homes from houses and apartments to backyard rooms, work quarters, squatter camps and informal settlements. The HH sample compares favorably with the South African statistics as 15 % lived in informal dwellings compared with national statistics of 13.6 - 14.1 %. The remainder lived in formal homes (flats and houses – 73 % HH versus SA- 71 %) and work quarters or backrooms (8 % HH versus 3.7 % Nationally) [19]. In terms of the rural/urban split, despite the one third of the South African population (38.3 %) living in rural areas [21], the HH sample, which is 7 % rural, compares favorably with Gauteng, Western Cape and KwaZulu Natal statistics as indicated in Fig. 1: Urban vs Rural breakdown by province for the HH sample (*n* = 532) in comparison to provincial population [20].

A further indicator of socio-economic status is access to the “Care Dependency Grant” that is offered to South African parents who have a child with a disability and whose income is below R151,000 per annum (equivalent to approximately $13,500 US) per individual (married or single). However information on this variable was missing in 39 % (*n* = 203) of the returns of those potentially eligible (*n* = 522) therefore was not used as a proxy for socio-economic status.

We explored the representativeness of the HH sample in terms of maternal education in comparison with official statistics on South African women [17]. The national comparator available was ‘women’ rather than ‘mothers’ and no information was found on women of child-bearing age alone. Therefore whilst the comparisons drawn are the best fit, they are not necessarily exact. Data were available on 504 out of 532 of the primary caregivers (which included birth mothers as well as foster or relative ‘mothers’ that were primary caregivers) in the HH data set and will be presented in four broad categories: mothers who had no schooling at all, those mothers who had some schooling (either primary or secondary schooling) but did not matriculate (i.e. graduated out of secondary school at 12^th^ grade level), those who matriculated from secondary school but did not study further, and those who had a post-matric qualification.

Of these 504 HH mothers, 100 % had some level of schooling unlike the national sample where 15 % of women in South Africa are reported to have no education. A similar number of HH mothers attended school but did not matriculate (215/504 = 42.7 %) and matriculated (220/504 = 43.6 %) in comparison to the national survey where 63.6 % of women in the national survey attended school but did not matriculate and only 13.8 % in the national survey achieved a matric. Finally 13 % of HH mothers had qualifications higher than Grade 12 level (*n* = 69) in comparison with only 5.3 % of women nationally.

### The relationship between newborn hearing screening, maternal suspicion and age of identification of deafness

**Fig. 2:**
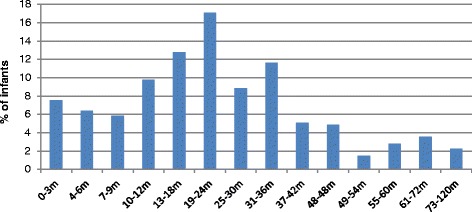
Age of identification (*n* = 532)

For this study group (sample) (*n* = 532), the median age of identification of deafness was 24 months (interquartile range (IQR) 12–36 m). 40 % of the infants were identified after 24 months (this includes 4 out of the 13 children with unilateral losses), missing the crucial first two years of the language development age, and less that 15 % of the infants were identified before 6 months of age, which is the gold standard age-range for the EHDI full spectrum of screening, diagnosis and the start of early intervention. Figure 2: Age of identification (*n* = 532).

**Fig. 3:**
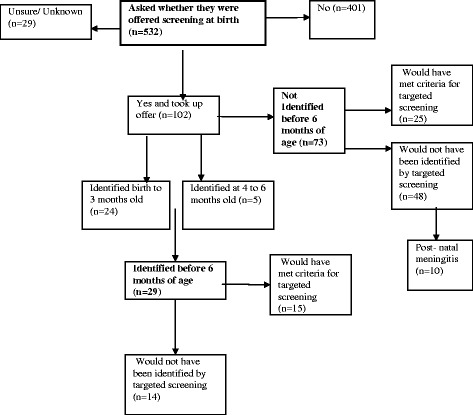
Outcomes for sub-sample of those offered newborn hearing screening in comparison with targeted screening criteria

Of the 532 infants, 102 (19 %) of their parents, 71 % of whom were from the public health sector, said they had been offered newborn hearing screening and had taken it up. Currently newborn hearing screening is offered in South Africa primarily by qualified audiologists or acousticians supervised by an audiologist. It always includes OAE screening and in some cases ABR but there is no national standard. Four hundred and one parents (75 %) stated that they had not been offered newborn hearing screening and 29 were not given information about it or reported being unsure (see Fig. 3). Of the 102 infants who were screened, the screen resulted in identification of deafness for 28 % (29/102 including 3 who had unilateral losses), 24 within the 0–3 month age bracket and a further 5 within the 4–6 month age bracket (including one child with a unilateral loss). Of those infants correctly identified as deaf through newborn hearing screening, half would have met the criteria for targeted screening (52 %) because of recognized risk factors [13], and half would not (48 %).

The high percentage of babies who were screened at birth and who did not meet the target of identification of deafness before the age of 6 months (*n* = 73, 71 %) and who were subsequently confirmed to be deaf, calls into question the justification for newborn hearing screening at centres where it was being practised. Whilst some of this late diagnosis might be accounted for by acquired hearing loss as a result of illness, accident or genetic origin, it is unlikely to account for nearly three quarters of the infants passing their newborn screen. Further investigation of the 73 who passed their screen and later were diagnosed as deaf shows only ten were known definitively to have had post-natal meningitis and 25/73 had high risk features at birth that would have met the criteria for targeted screening in any case i.e. more likely to have a newborn hearing loss. These high risk infants included 8 who had been in NICU for more than 48 h, 3 babies with craniofacial abnormalities and/or atresia, five infants whose mothers had in-utero infections (CMV, HIV or Rubella) and five who had family members with a hearing loss. Ten of these 25 babies (40 %) had multiple risk factors.

We summarise these results in Fig. 3: Outcomes for sub-sample of those offered newborn hearing screening in comparison with targeted screening criteria.

Given these numbers of ‘missed’ high risk infants and their subsequent late identification, as well as the large number of infants not routinely offered newborn hearing screening, we now explore maternal suspicion of hearing loss in their infant. This item was added to the registration form in the 4^th^ year of the HI HOPES programme and of the 305 infants who joined the programme in years 4 and 5, 247 mothers (81 %) said they had a suspicion that their child had a hearing loss prior to confirmation/diagnosis (see Table 2). This includes only two mothers of children later diagnosed with a unilateral hearing loss.

**Table 2:** Summary of median and mean age of suspicion, identification and delay by health sector

		Total		Private health		Public health	
		*N* = 247		*N* = 28		*N* = 219	
Age of suspicion (months)	Mean/SD	21.2	14.6	21.6	18.2	21.2	14.1
	Median/IQR	18	11–24	12.5	8–33	20	12–24
Age of identification (months)	Mean/SD	32	20.4	27.1	19.7	32.6	20.5
	Median/IQR	28	18–40	22	12–35.5	28	19–41
Delay to identification (months)	Mean/SD	10.8	13.3	5.5	7.4	11.5	13.8
	Median/IQR	6	0–15	2	0–8.5	7	0–15

We compared age of the child at maternal suspicion of hearing loss (henceforth referred to as age of suspicion) in the public health (*n* = 219) and private health (*n* = 28) sectors and no significant difference was observed (Wilcoxon rank sum test; *p* = 0.50). Overall, the median age of suspicion was 18 months (IQR 11–24 m). When comparing the age of identification of these infants, there was also no significant difference in the median age of identification between the private and public health sectors (*p* = 0.10). The median age of identification was 28 months (IQR 18–40 m).

However, the median delay between age of suspicion and age of identification was significantly longer in the public sector (7 m; IQR 0–15 m) compared to the private sector (2 m; IQR 0–8.5 m) (*p* = 0.035). This could either indicate that the private health sector is more open to responding to and addressing maternal suspicion or those parents in the private health sector are more assertive when sharing their suspicions. It may also be indicative of the disparity between both the resources and staffing of the public and private health sectors. See Table 2: Summary of median and mean age of suspicion, identification and delay by health sector.

**Fig. 4:**
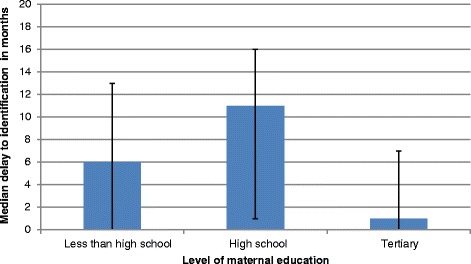
Delay between age of suspicion and age of identification (*n* = 247)

We examined the relationship between level of maternal education and age of suspicion of their child’s deafness. Of the 247 mothers, maternal education information was available for 235, where 118 (47.8 %) mothers indicated they attended school though did not matriculate, 99 (40.1 %) had a matric certificate and 19 (7.7 %) indicated they had a post-matric qualification. There was no significant difference in the median age of suspicion and maternal level of education (Kruskal-Wallis test; *p* = 0.35), or the median age of identification (*p* = 0.89). However, the median delay from suspicion to identification was shorter for those with tertiary education (48 % private health care; 52 % public health care), compared to the other two groups (*p* = 0.015), as illustrated in Fig. 4: Delay between age of suspicion and age of identification (*n* = 247).

**Fig. 5:**
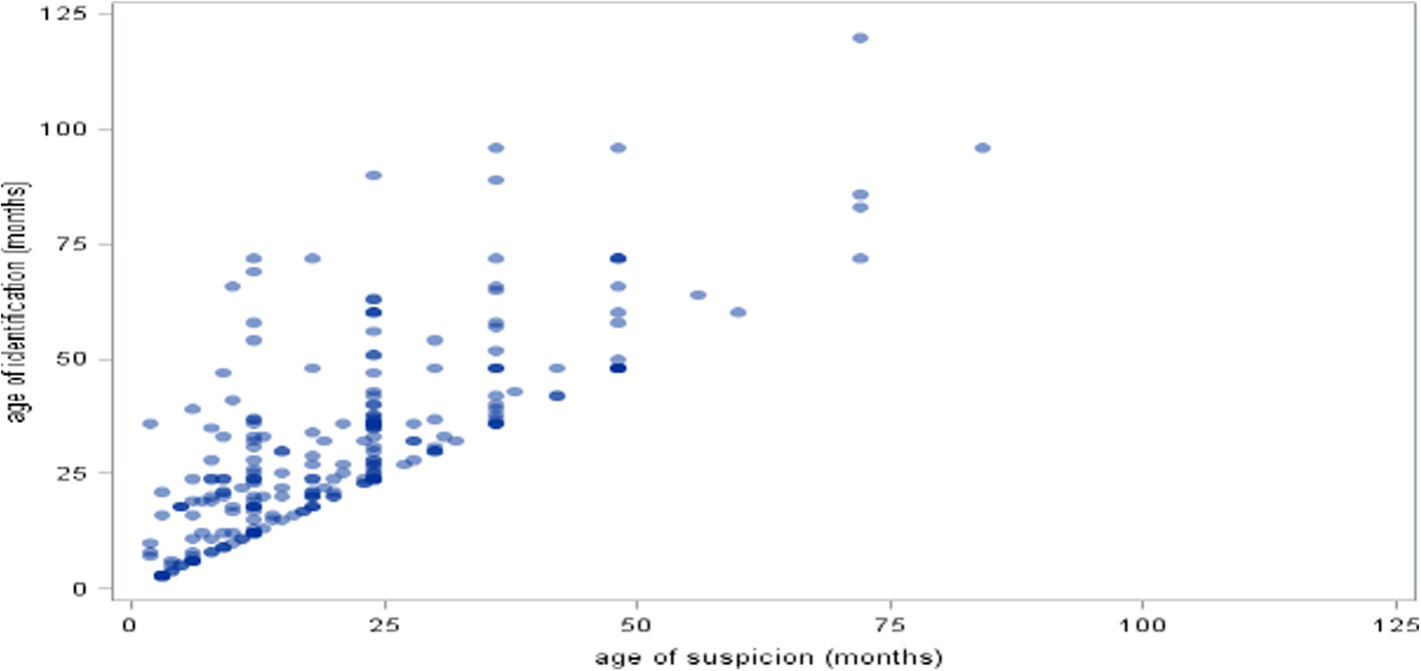
The relationship between age of maternal suspicion and age of identification of infant hearing loss (*n* = 247)

We examined the relationship between age of suspicion and age of identification. For this sample of 247, only 23 infants (9 %) had newborn hearing screening, only one of whom was identified as deaf in the first 3 months of life. 224 (90.6 %) infants were not screened at birth. Figure 5 provides the overall plot of the age of identification in relation to the age of maternal suspicion, where the darker areas reflect the higher frequency of occurrences. These coincide with key child developmental milestones of 8 months, 12 months, 18 months, 24 months and 36 months of age where differences between expected development and actual development are likely to be more noticeable to parents. Figure 5: The relationship between age of maternal suspicion and age of identification of infant hearing loss (*n* = 247).

The plot demonstrates large differences in age of identification for the same key points of maternal suspicion with many mothers being suspicious prior to much later identification. We therefore investigated the delay between age of suspicion and age of identification and found no significant correlation (*p* = 0.32). In other words, an earlier age of suspicion did not predict an earlier identification of infant hearing loss.

We investigated further the delay between age of suspicion and age of identification in the sample of 247 but excluding the 23 infants known to have received newborn hearing screening and the 47 infants known to have contracted meningitis in infancy i.e. they would reasonably have been expected to become deaf after the age at which newborn hearing screening would have been carried out. Of the remaining 177, 51 mothers’ suspicion led to an immediate identification of their child’s hearing loss, where the age of suspicion became the age of identification. 41 out of 51 of these infants were in the public health care sector. Eleven of these infants were identified before 6 months of age (9 in the public health care sector), 3 of whom (all in public health) were identified before 3 months of age.

## Discussion

In low and middle income countries, health policy and practice developments are hampered not just by scarcity of resources but by lack of research evidence to support decisions about the prioritization of those scarce resources and the targeting of effort to ensure maximum impact. Whilst internationally universal newborn hearing screening resulting in early identification of deafness is clearly identified as best practice for deaf children’s optimal development, evidence to support its implementation in South Africa has been scarce. Small scale studies might demonstrate a degree of feasibility of EHDI in specific circumstances for particular populations but have been unable to demonstrate broader relevance. Cohorts of deaf children studied have been small and not necessarily representative of the diversity of South Africa’s population [1, 4, 5].

In this study, we have established the characteristics and extent of representativeness of the largest cohort, as far as we are aware, of deaf children under the age of 6 in Africa who have been studied longitudinally. In subsequent papers we will address their language development in relation to their age of identification and extent of intervention uptake. In this paper we have demonstrated that the HH cohort can be considered representative in terms of the racial profile of the three provinces from which they are drawn and the distribution of private and public health care use. The sample, however, displays a higher level of maternal education in comparison with that of women nationally and indicators of socio-economic status suggest the sample is skewed slightly toward lower income families. The HH sample (*n* = 532) therefore provides a substantial basis on which to explore a range of issues concerning deaf children and their families with some confidence of the relevance and generalizability of the results to the general population in South Africa and deaf children nationally.

The median age of identification in this sample (*n* = 532) of 24 months, with 40 % identified after 24 months, far exceeds what is now regarded internationally as optimal for deaf children, before 3 months old, but that standard is in the context of universal newborn hearing screening. As our results also show, access to newborn hearing screening in the context of South Africa is very limited. Furthermore, in the HH sample of deaf children, very few infants who underwent newborn hearing screening were identified as deaf as a result of it (29/102) calling into question its sensitivity and specificity where it was being practised. This result is not explicable by later onset deafness. Furthermore, of the 29 identified, 15 would have met the criteria for targeted screening in any case. These results reinforce the call for newborn hearing screening, where it is being offered in South Africa, to be of a very high quality. However, they also potentially support calls for whether a programme of well executed targeted screening might be more appropriate on a universal basis [12]. In a socio-economic context where competing priorities in health care such as TB and HIV are fundamentally concerned with survival, the high cost of implementing a universal newborn hearing screening programme might not be justifiable.

However, our data also opens up an additional perspective seeking to ensure earliest possible identification of deafness; that of believing and acting on maternal suspicion. In our sample of 247 (out of 305) mothers who expressed a suspicion that their child might have a hearing loss, the median age of suspicion was 18 months compared with the median age of identification of 28 months, with no significant differences found between those using the private or public health care systems. A difference of 10 months in a developing infant’s life is highly important particularly as 18 months of age usually marks the beginning of the vocabulary spurt during which not just the number but the rate of acquisition of new lexical items increases dramatically [9]. Impediments in access to language during the period are particularly problematic for deaf children with regard to their later language development [6, 13]. We found no significant relationship between maternal level of education and age of suspicion of an infant’s deafness indicating that suspicion is more likely to be associated with the maternal/child relationship, borne out by the most frequently occurring ages of suspicion coinciding with key infant developmental milestones. Acting on maternal suspicion thus offers an additional gateway to earlier identification.

However, we found no statistically significant relationship between the age of the infant at maternal suspicion of hearing loss and age of identification demonstrating that earlier age of suspicion did not lead to earlier age of identification. Furthermore, the median delay between age of suspicion and age of identification was statistically significantly longer in the public (majority) compared with the private (minority) health care system. However, the median delay from suspicion to identification was shorter for those with tertiary education, compared to those with lesser educational levels (*p* = 0.015). Equivalent numbers in private and public sector health care users had tertiary education meaning indicating that it is level of education and not private versus public health care services that makes a difference.

From these results it is not possible to conclude whether it is the health care system that is tardy in its response to maternal suspicion, or whether it is mothers (and their families) who are not encouraged to act on their suspicions, or reluctant to do so, but the results do show the potential for considerably reducing the age of identification of infant hearing loss if maternal suspicion can be acted on in a timely manner. This is currently not happening. In the HH sample (*n* = 247), if the screened infants and those with meningitis (therefore likely to have developed deafness later in any case) are removed, in only 22.7 % (*n* = 51) of cases did age of maternal suspicion coincide with age of identification with only 11 being identified before the age of 6 months. However, of those 51, 41 were drawn from the public health care system refuting any assumption that it is the private health care system of the minority that is likely to act more efficiently or swiftly in response to maternal suspicion.

## Conclusion

Low and middle income countries are quite rightly seeking cost-effective solutions to major health problems and social divisions arising from inequitably distributed life opportunities and resources. Within this range of health challenges needing to be met hearing loss is not often seen as a priority and within an already overburdened health system, funding that is available in developing countries is distributed to the most pressing and widely spread issues such as HIV Aids, TB and sanitation to name just a few.

Despite this, developing countries have begun to acknowledge the importance of late identified hearing loss [31] and its significant impact on the lives of children in the long-term and therefore on the state because of the subsequent financial burdens. Our data point to the interim advantages of introducing more comprehensive targeted screening programmes and fundamentally the importance of acting swiftly on maternal suspicion of deafness as a strategy to improve the life chances of deaf children and their families until the case for universal newborn hearing screening in South Africa is clearly justified. Believing mothers costs nothing.

## Abbreviations

CI: cochlear implant
CMV: cytomegalovirus
EHDI: early hearing detection and intervention
ENT: ear nose and throat (surgeon)
GT: Gauteng (Province)
HH: HI HOPES
HIV: human immunodeficiency virus
IQR: inter quartile range
JCIH: Joint Commission on Infant Hearing
KZN: Kwa Zulu Natal (Province)
NICU: Neonatal Intensive Care Unit
R: rand
SA: South Africa
SAIRR: South African Institute of Race Relations
TB: tuberculosis
WC: The Western Cape (Province)
WHO: World Health Organisation

## Footnotes

1 Deafness is defined as any type or level of hearing loss, including both uni- and bilateral hearing losses.
